# Signal, Transduction, and the Hematopoietic Stem Cell

**DOI:** 10.5041/RMMJ.10167

**Published:** 2014-10-29

**Authors:** Igal Louria-Hayon

**Affiliations:** 1 Department of Hematology, Rambam Health Care Campus, Haifa, Israel; 2 Department of Biotechnology, Hadassah Academic College, Jerusalem, Israel

**Keywords:** Differentiation, HSC communication, niche, self-renewal, signal transduction

## Abstract

The hematopoietic stem cell (HSC) is a unique cell positioned highest in the hematopoietic hierarchical system. The HSC has the ability to stay in quiescence, to self-renew, or to differentiate and generate all lineages of blood cells. The path to be actualized is influenced by signals that derive from the cell’s microenvironment, which activate molecular pathways inside the cell. Signaling pathways are commonly organized through inducible protein–protein interactions, mediated by adaptor proteins that link activated receptors to cytoplasmic effectors. This review will focus on the signaling molecules and how they work in concert to determine the HSC’s fate.

## THE SIGNAL

In the *Tractatus Logico-Philosophicus*, Ludwig Wittgenstein stated that “the limits of my language are the limits of my world.”^1^ This assertion of Wittgenstein’s may guide us while we try to decipher the sophisticated pattern of cell communication. To date, we have accumulative data, which supports the notion that cells do communicate during their existence. Remarkably, in mammalian cells, there is evidence for cell communication, even in its extreme form as in the case of cancer cells,^2^ cells in quiescence, or during senescence and apoptosis.^3^ Thus, we may hypothesize that every cell in a multicellular organism has the ability to communicate. Similar to any other language, the molecular message has to be accurate. Therefore, in the cell, the communication is tightly regulated, and mistakes may lead to dismal outcomes. An incorrect message processing may promote a severe disease.

Signaling is frequently a reciprocal process, in which different populations of cells exchange molecular signals to induce complex cellular architectures. Thus, one cell type can produce a soluble factor that promotes the differentiation, function, and survival of a target cell. Over the past five decades, many forms of signal elements were discovered and were termed as growth factors, neurotransmitters, interleukins, cytokines, chemokines, and hormones. These factors are all signals, which are produced under specific physiological conditions in order to interact and activate a specific receptor on the surface of target cells. Thus, messages are delivered in order to stimulate cell activities in relation to the organism’s physiological condition and requirements. Accordingly, signaling is essential for the unification of a single cell with its environment, and in the case of the multicellular organism to bond the single cell with the whole.

## THE TRANSDUCTION

In Wittgenstein’s *Philosophical Investigations*, language is released from its confining stasis and takes part in the process of becoming, since “Language is itself the vehicle of thought” (remarks 329).^4^ Such communication processes are called transduction when referring to the living organism. Transduction is the processing sequence of the signal. The root “duce,” meaning “to lead” in Latin, marks the movement of a message from the ligand-activated receptor into the cell. Thus, the signal becomes synonymous with the message, and transduction correlates with the understanding process. Such processes evolved into sophisticated internal cell machineries characterized by unique formations.^5^ The first response to the activated receptor is the recruitment of signaling molecules. These regulatory proteins are frequently constructed in a cassette-like fashion from one or more domains that mediate molecular interactions or have enzymatic activity.^6^,^7^ In the evolutionary history of living organisms a diverse array of protein domains has evolved to interact with a specific sequence on a target protein leading to a communication mechanism for signaling molecules in a network influenced by activated receptors.^8^ Such domains can be viewed as portable units of biological function that provide a mechanism for the evolution of new cellular activities and new molecular connections within the cells.^9^,^10^ Interaction domains can target proteins to a specific subcellular location, provide a means for recognition of protein posttranslational modification or chemical second messengers, establish the formation of multiprotein signaling complexes, and control conformation, activity, and substrate specificity of enzymes.^11^ In signal transduction, enzymes (kinases, for example) often generate modified amino acids on their substrates that are then recognized by interaction modules. An example of such a molecular communication system is the phosphor-tyrosine (pTyr)-Src homology 2 (SH2) domain-based signal transduction.^12^ The binding of a ligand to the extracellular domain of a receptor such as tyrosine kinase (RTK) induces dimerization of the receptor, leading to the activation of the intrinsic tyrosine kinase and intermolecular autophosphorylation.^13^ This induces a physical association between SH2-containing cytoplasmic signaling proteins and the activated receptor. The SH2 domains directly recognize phosphorylated tyrosine residues; they also have independent binding sites for residues surrounding the phosphotyrosine within a polypeptide chain. Receptor phosphorylation therefore creates a SH2 binding site on the receptor; the receptor sequences flanking the phosphotyrosine dictate which particular SH2 domains will bind with high affinity to which tyrosine-phosphorylated receptor.^14^ This mechanism can induce cells to proliferate, migrate, and differentiate, or, in cases of mutations, can result in disorders which contribute to cancer development.^15^ Hence, tyrosine-based signaling is of greatest interest both for understanding the regulation of the normal cell and for defining the alterations in signal transduction that occur in cells with aberrant tyrosine kinase activity. The principles established for phosphorylation-dependent interactions have recently been extended to other forms of posttranslational modifications because N-glycosylation, acetylation, methylation, and ubiquitination of proteins can all function like phosphorylation to control modular protein interactions.^16^–^18^

## SIGNAL TRANSDUCTION AND HEMATOPOIETIC STEM CELLS

“Everyday language is a part of the human organism and is no less complicated than it.”^1^ The inherent role of communication in the organism manifests distinctively in the hematopoietic stem cell (HSC). The HSC is a unique cell positioned highest in the hematopoietic hierarchical system. The HSC has the ability to stay in quiescence (cell cycle arrest in G0), to self-renew, or to differentiate and generate all lineages of blood cells.^19^–^21^ Since the pioneering work of James Till and Ernest McCulloch who showed that single cells could yield multilineage descendants while preserving the multipotency of the mother cells,^22^ the HSC was the focus of studies aiming to define molecular and signaling pathways and how they work in concert to determine their phenotypic and functional characterization.^23^ For more than five decades, the unique capacities of HSCs have been applied to regenerate the hematopoietic system in the procedure of bone marrow transplantation.^24^ Thus the HSC represents an exclusive sample for signaling, which governs the cell’s end and, sequentially, that of the organism as a whole.

### Signal Transduction in the HSC-niche Synapse

**Figure 1.**
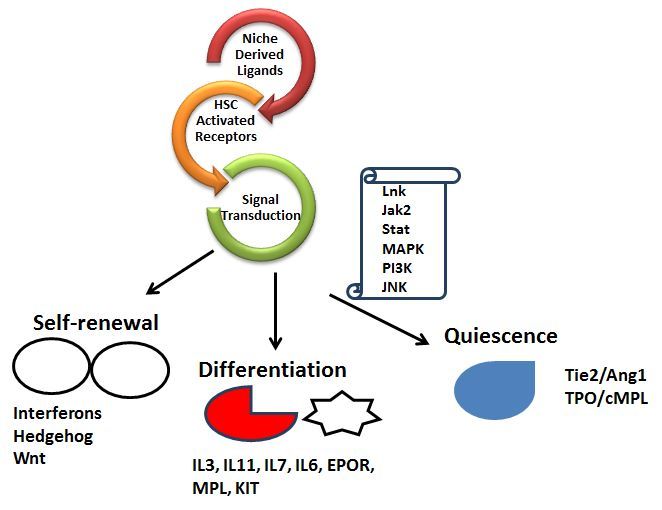
**Ligands Derived from the HSC Microenvironment Induce Receptors on the HSC to Activate Signal Molecules Inside the Cell which Determine the Cell’s Fate.**

A wide range of experimental evidence suggests that the function of the HSC to retain both self-renewal and multilineage differentiation after transplantation is dependent on signals that derive from the HSC microenvironment (also termed the HSC niche)^25^ (Figure 1). Postnatally, the bone marrow is the primary site for HSC maintenance and hematopoiesis. Early studies formulating the osteoblastic niche theory showed that primitive cells tended to localize towards the endosteal margins of the bone, leading to the hypothesis that the bone environment regulates hematopoiesis.^26^ Furthermore, largely mesenchymal “stromal” cell cultures and osteoblast differentiated in culture from human bone marrow stromal cells could maintain primitive hematopoietic cells *ex vivo*.^27^ However, the “osteoblastic” niche theory was challenged by studies showing that osteoblasts depleted by *Bgn* deficiency or osteoblastic cells depleted by treatment with ganciclovir had no acute effect on HSC frequency.^28^,^29^ Current data suggest that there are specialized niches for distinct types of hematopoietic stem and progenitor cells, and each niche may be created by multiple cell types, which in turn influences the HSC.^30^ The HSCs are found mainly adjacent to sinusoid throughout the bone marrow, where endothelial cells and mesenchymal stromal cells promote HSC maintenance by producing SCF, CXCL12, IL-6, RANKL, and Jagged1.^31^ Thus, while osteoblasts may have a secondary influence on the HSC, both endothelial cells and mesenchymal stromal cells produce factors which can potentially directly interact with the HSC and regulate its activity.

A complex molecular cross-talk between the HSC and the niche controls the balance between self-renewal and differentiation. It is commonly assumed that this balance is achieved by asymmetrical cell division through which one daughter cell maintains the stem cell identity and the other becomes differentiated.^32^ This asymmetry can be achieved by environmental signals, which create pro-differentiation or pro-renewal environments.^33^

## QUIESCENCE

Adults’ HSCs are mostly quiescent with approximately 95 % in the G0 phase of the cell cycle. The maintenance of a dormant pool of non-cycling and metabolically inactive HSCs was suggested as a protective mechanism against exhaustion of limited self-renewal potential.^34^,^35^ In its quiescent form, the maintenance of HSCs is tightly regulated by signals from the environment. Ang1 is a ligand for the Tie2/Tek receptor which is expressed by endothelial cells, osteoblast cells, and HSCs.^36^ Upon interaction, the Tie2/Ang1 complex induces signaling, which activates B1-integrin and N-cadherin, thus promoting HSC dormancy *in vivo*.^36^ Tie2 is a tyrosine kinase receptor that in endothelial cells was demonstrated to recruit signaling proteins such as Grb7, Grb2, Shp2, and Shc1, which activate signaling pathways such as pi3K and MAP kinases.^37^ However, which of these pathways promote quiescence of HSCs is yet to be revealed.

Another cytokine that maintains quiescent HSCs is thrombopoietin (TPO), the ligand for c-MPL receptor.^38^ Various tissues, which participate in hematopoiesis, expressing c-MPL, include bone marrow, spleen, and fetal liver.^39^ Long-Term-HSCs (LT-HSCs) expressing c-MPL receptor were found in correlation to cell cycle quiescence and are closely associated with TPO-producing osteoblastic cells in the bone marrow.^38^ The binding of TPO to c-MPL induces receptor homodimerization and recruitment of Janus kinase 2 (JAK2), which phosphorylates tyrosine residues within the receptor itself.^40^ The phosphorylated residues Tyr625 and Tyr630 stimulate the downstream cascade signal transducer and activator of transcription (STAT), phosphoinositide 3-kinase (PI3K), and mitogen-activated protein kinase (MAPKs).^41^ Additionally, the adaptor protein Lnk was revealed as an inhibitor of JAK2 in HSCs following TPO stimulation,^42^–^45^ and as a regulator of the resistance capacity of normal and cancerous HSCs in response to irradiation treatment.^46^ Thus, the MPL/JAK2/Lnk pathway can be concluded to be a gatekeeper for HSC quiescence.

Signaling by TGF-β/Smad was also proposed to participate in maintaining HSC quiescence.^47^ *In vitro* culture studies revealed the inhibitory effect of TGF-β on HSC proliferation and that the neutralization of TGF-β *in vitro* released HSC from quiescence.^48^ Several mechanisms were suggested for the inhibitory effect of TGF-β on HSC proliferation, including alteration in cytokine receptor expression and stimulation of cyclin-dependent kinase inhibitors such as p21 and p27.^49^ However, conditional knockout mouse strategies aiming to elucidate the direct effect of TGF-β signaling on the HSC *in vivo* revealed normal self-renewal and no effect on HSC proliferation.^50^ Therefore, to date, Tie2/Ang1 and MPL/TPO/Lnk signaling pathways are the most critical pathways that regulate HSC quiescence and thus are the ideal target for modifications of HSC quiescence.

## SELF-RENEWAL

The ability of the HSC to self-renew is regulated by an exclusive array of signaling, e.g. interferons, Hedgehog, and Wnt.^51^ Recent evidence strongly suggests that Wnt signaling has an important regulatory role in hematopoietic progenitors/stem cells during both fetal and adult development.^52^ Wnt proteins represent a family of secreted signaling molecules that are expressed in diverse tissues. Wnts act by binding to two types of receptor molecules. One is the Frizzled family of seven-pass transmembrane proteins, which contain a cysteine-rich extracellular domain that binds to Wnt proteins.^53^ The second is a subset of the low-density lipoprotein receptor-related protein (or the LRP) family, specifically, LRP-5 and LRP-6.^54^–^56^ It was demonstrated that both LRP-5/6 and Frizzled are required to activate functionally the downstream components of the canonical pathway.^57^ In the absence of a Wnt signal, β-catenin is subjected to rapid degradation via association with a protein complex that includes the scaffold protein Axin and the serine/threonine kinase, glycogen synthase kinase-3 beta (GSK-3). In this complex, β-catenin is phosphorylated at its NH_2_ terminus by GSK-3 and thus targeted for ubiquitination and degradation by proteasomes.^52^ Axin acts as a scaffold to enhance significantly the ability of GSK-3 to phosphorylate catenin. The binding of Wnt proteins to their receptors inhibits phosphorylation of β-catenin by GSK-3. This prevents β-catenin’s degradation and results in stabilization and translocation of β-catenin to the nucleus, where it binds to members of the LEF/T-cell factor (TCF) family of transcription factors.^52^ The LEF/TCF proteins are normally associated with the transcriptional repressor, Groucho, which suppresses their activity and represses gene transcription.^58^ The binding of β-catenin relieves this repression and allows LEF/TCF factors to induce expression to the appropriate target genes.^52^ Typically, the downstream effectors for transcriptional activation target genes are FGF20, DKK1, WISP1, MYC, and CCDN1.^31^ The second pathway, “non-canonical Wnt signal,” does not involve β-catenin activity and regulates polarization of the cells and Ca^2+^ concentration to activate protein kinase C and calmodulin-dependent kinase.^59^

During fetal hematopoiesis, Wnt proteins—specifically, Wnt5A and Wnt10B—are expressed in the embryo’s yolk sac and the fetal liver. It was shown that conditioned media containing Wnt1, Wnt5A, or Wnt10B stimulate the stem cell growth factor.^60^ Moreover, the exposure of CD34^+^Lin^−^ human hematopoietic progenitors to Wnt5A, which was found to be expressed in these precursors, promoted the expansion of undifferentiated progenitors in the presence of stromal cells. Overexpression of activated β-catenin in long-term cultures of HSCs induced cells to enter the cell cycle and grow in long-term cultures.^61^ These expanded HSCs also retained the functional characteristics of HSCs, following transplant into allelically distinct irradiated mice. Inhibition of Wnt signaling in HSCs by overexpression of the inhibitor of canonical Wnt signaling, Dickkopf1 (Dkk1), resulted in the induction of cell cycling and reduction in the repopulating ability of transplanted induction mice.^62^ In addition, the inhibitor of GSK-3β delays cell cycle progression of CB-CD34^+^ cells, and promotes *ex vivo*-expanded HSCs.^63^ These studies demonstrate that Wnt signaling is important in the maintenance and self-renewal of hematopoietic stem and progenitor cells.

Type I (α and β) and type II (γ) interferons (IFNs) belong to a family of cytokines which orchestrate numerous immunological and cellular processes such as cell motility, cell proliferation, antibody response, graft rejection, natural killer cell recruitment, and macrophage activation.^64^ Type I and type II IFNs signal through distinct but related pathways. Interferon receptors bind to JAKs and to signal transducers and the activator of transcription (STATs), thus having the potential to activate various molecular signaling pathways.^65^ The main pathway of response to IFNα/β requires two receptor subunits, two JAKs, two STATs, and the interferon response factor (IRF) family transcription factor p48. Dimerization of the receptor leads to initiation of the tyrosine-phosphorylated cascade, where JAK1 phosphorylates and activates Tyk2, which cross-phosphorylates JAK1, resulting in activation and sequential phosphorylation of Y466 of IFNAR1, Y690 of STAT2, and Y701 of STAT1.^66^ Upon IFN receptor activation the SH2 domain of STAT2 interacts with pY466 of IFNAR1 followed by the phosphorylation of both STATs and the dissociation of the heterodimer from the receptor.^66^ The activated STATs then transport to the nucleus where they bind to specific DNA sequences and stimulate transcription. Most cell types stop proliferation in response to IFNα.^67^ However, *in vivo* studies in mouse models revealed that high levels of IFNα induce HSC proliferation.^68^,^69^ Thus, IFNα signaling must be fundamentally different in the HSC. Mice that were genetically deficient for a negative regulator of type I interferon signaling, interferon response factor-2 (IRF2), exhibited enhanced proliferation of HSCs which impairs the ability to repopulate the bone marrow of irradiated mice,^68^ indicating that this cell population was no longer fully functional. In addition, high levels of IFNα directly induced wild-type HSCs to exit quiescence and transiently proliferate *in vivo*.^68^ In spite of the HSC unique response to IFNα, it was shown that STAT1 is required for IFNα-mediated exit from dormancy,^69^ indicating that the unusual effect on proliferation is mediated by a canonical IFN signaling component. Indeed, these studies show that IFNα induces HSC proliferation; however, it is yet to be determined how the HSC uniquely interprets canonical IFNα signaling. Interestingly, in contrast to IFNα, it was demonstrated that IFNγ negatively modulates self-renewal of the human HSC^70^ and impairs proliferation of HSC in mice.^71^ Thus in the HSC, the role of IFN type I signaling is distinctive from the role of IFN type II.

The Hedgehog (Hh) pathway is a highly conserved developmental pathway which regulates the proliferation, migration, and differentiation of cells during development.^72^ It is typically active during development, but silenced in adult tissues, except during tissue regeneration and injury repair.^73^ The Hedgehog (Hh) ligand binds to the transmembrane receptor Patched (Ptc) and subsequently allows the signaling function of a second transmembrane protein, Smoothened (Smo), to be activated. Hedgehog is proposed as a negative regulator of the HSC quiescence.^74^ It was demonstrated that constitutive activation of the Hh signaling pathway in Ptc heterozygous (Ptc-1^+/−^) mice resulted in induction of cell cycling and expansion of primitive bone marrow hematopoietic cells.^74^ Deletion of Smo *in utero* in transgenic mice supports this hypothesis and demonstrates an impaired stem cell self-renewal and inhibition in the engraftment activity of the HSC.^75^ Furthermore, the common downstream positive effector of Hh signaling, Gli1, has been shown to play a critical role in normal and stress hematopoiesis.^76^ However, in some studies the conditional loss of Smo within adult HSCs is dispensable for hematopoiesis.^77^,^78^ These conflicts might be due to the difference between the mouse model and the conditional system used to impair Hh signaling.

## DIFFERENTIATION

The main purpose of the HSC is to maintain and keep the hematopoietic system functioning under normal or stress conditions; therefore, the HSC has the capacity to differentiate and generate all blood cells in response to environmental signals which deliver the organism’s requirements under specific conditions. Hematopoietic stem cells were studied extensively in order to identify the molecular players and routes, which distinguish differentiation from self-renewal.

A wide array of receptors, including IL-3R, IL-11R, IL-7R, IL-6R, EPOR, MPL, and KIT, induce differentiation.^35^ This is due to a network of signaling molecules such as MAPKs pathways which can determine the HSC’s fate towards differentiation.^79^

The MAPKs are a family of serine/threonine kinases that play an essential role in signal transduction after receptor stimulation. Three major groups of MAPKs have been characterized in mammals, including ERKs, JNKs, and P38MAPK.^80^

The kinases ERK1 and ERK2, also known as p44^MAPK^ and p42^MAPK^, respectively, were identified as growth factor-stimulated protein kinases phosphorylating MAP-2 and myelin basic protein.^81^ They have more than 80% aa sequence similarity and can be activated by a wide variety of stimuli, including growth factors, serum, and cytokines.^80^ Upon activation, ERK1/2 phosphorylate and regulate the activity of cytoplasmic molecules and nuclear proteins, which in turn can control gene expression.^81^

Studies on differentiation-competent cell lines revealed the importance of the ERK signaling module in regulating myeloid, erythroid, and megakaryocyte differentiation.^79^ Furthermore, ERK1/2-mediated phosphorylation of the C/EBPα transcription factor on serine residue 21 was found to regulate negatively the activity of C/EBPα and its ability to induce neutrophil differentiation.^82^ The leucine zipper transcription factor C/EBPα plays a critical role in regulating myelopoiesis, and mice deficient for C/EBPα lack mature granulocytes and accumulate immature myeloblasts in the bone marrow. The underlying molecular mechanism was found to involve regulation of expression of a variety of cell-cycle-modulating proteins, including c-myc, c-fos, p21CIP1, cyclin D1, and cyclin D3. In addition ERK MAPK signaling were shown be involved in the regulation of early myeloid commitment of the HSC.^83^ Taken together, these results suggest that activation of the ERK pathway is required for normal hematopoiesis.

The JNK MAPKs are also known as stress-activated protein kinases. Downstream substrates of JNKs include the transcription factors c-Jun, Elk-1, p53, ATF-2, and NFAT; MAPs; and proapoptotic Bcl-2 family members, including Bid, Bax, and Bim.^84^ Although JNK proteins were first identified as kinases that were activated by a stress- and apoptosis-inducing agent, JNKs are now known to be activated by a variety of growth factors that regulate proliferation, differentiation, and survival of hematopoietic cells, including EPO and SCF, as well as TPO, IL-3, and GM-CSF.^79^ Furthermore, several studies focusing on JNK function in hematopoietic cell systems revealed the importance of the JNK signaling module in regulating erythropoiesis.^85^ Inhibition of JNK activity in primary mouse bone marrow cells reduced the number of burst-forming unit–erythroid (BFU-E), whereas the more differentiated colony-forming unit–erythroid (CFU-E) were not affected. Moreover, it has been reported that disruption of the upstream JNK regulator MEKK1 causes embryonic death as a result of defects in erythrocyte differentiation, further indicating that the MEKK1–JNK signaling pathway is indeed essential for erythropoiesis.^86^ In addition, JNK1 was demonstrated to interact physically with the DNA-binding domain (DBD) of C/EBPα *in vitro* and *in vivo*.^87^ These studies demonstrate the importance of JNK signaling in the regulation of erythropoiesis and myelopoiesis.

## PERSPECTIVES

Understanding the signaling pathways that determine HSC fate is important for the success of a wide array of medicinal applications. These include HSC transplantation and cancer treatments, and may help refresh treatments strategies for auto-immune diseases as well as viral and bacterial infections. However, in spite of the vast number of studies focusing on the HSC, since the first successful bone marrow transplantation five decades ago, minor progress has been seen in the application of HSC studies. The reason may be found in the rarity of the HSC, which severely limits our ability to study their biochemistry. This also explains the nature of most studies conducted on HSCs, which mainly describe the phenomena and not the mechanisms—a trajectory that delays our ability to apply knowledge to practice. The prospective of HSCs in medicine is clear. While the potential to create red blood cells or any other type of blood cells, or the potential to expand HSCs, is driving the imagination of the drug industries as well as curious basic scientists to its limits, today it is clear that only a solid biochemical effort may bridge the gap from potency to concrete application.

The persistent study of the HSC is progressing forward, step by step, or gene by gene, via genetically modified mouse models. These investigations reveal the complexity of pathways and protein networks, which determine the HSC’s ends in self-renewal, differentiation, and quiescence. Studies have strikingly demonstrated that at times one pathway is involved in all three fates of the HSC, leading to the assertion that the physiological condition advancing toward the HSC’s final fate does not reside in a single route, but within a complex of interactions between several signaling pathways, and within a web of protein clusters. To date, we know that, downstream to activated receptors, protein clusters, which contain kinases, phosphatases, and adaptor proteins, are formed in order to activate transcription factors and sets of genes, which will govern the HSC’s fate. However, in order to uncover these intricate interactions of complexes and proteins which lead to the final destiny of the HSC, we have to transform our research strategies and invest our efforts in developing technologies which can reveal these types of complexes. Thus, in addition to genetic profiling of normal and cancerous HSCs,^88^ it is necessary to develop capacities to apply mass spectrometry technology in HSC research. This may reveal the protein networks which are formed in response to receptor induction towards differentiation,^89^,^90^ or in special cases such as blood diseases and malignancies during pregnancy.^91^,^92^ Finally, another strategy, which is applied in our lab, aims to understand protein interactions in the HSC via the case of adaptor proteins, which are known to be in the hub of cell signaling, and which are central to the protein complexes and protein webs. We believe that this strategy reveals which protein clusters determine the HSC’s fate.

## Abbreviations:

BFU-E: burst-forming unit–erythroid;
CFU-E: colony-forming unit–erythroid;
DBD: DNA-binding domain;
GSK-3: glycogen synthase kinase-3 beta;
Hh: Hedgehog;
HSC: hematopoietic stem cell;
IFNs: interferons;
IRF2: interferon response factor-2;
JAK2: Janus kinase 2;
RTK: tyrosine kinase;
SH2: Src homology 2;
STAT: signal transduction and transcription;
TCF: T-cell factor;
TPO: thrombopoietin.
